# Color discrimination repetition distorts color representations

**DOI:** 10.1038/s41598-024-60283-4

**Published:** 2024-04-26

**Authors:** Suzuha Horiuchi, Takehiro Nagai

**Affiliations:** Department of Information and Communications Engineering, Tokyo Institute of Technology, 4259-G2-1 Nagatsuta-Cho, Midori-Ku, Yokohama, 226-8502 Japan

**Keywords:** Psychology, Human behaviour

## Abstract

Perceptual learning is the improvement of perceptual performance after repeated practice on a perceptual task. Studies on perceptual learning in color vision are limited. In this study, we measured the impact of color discrimination repetitions at a specific base color on color perception for entire hues. Participants performed five sessions of color discrimination training (200 or 300 trials per session) over five days, at colors on either the negative or positive direction of the L-M color axis, based on group assignment. We administered three color perception assessments (unique hues, color category boundaries, and color appearance) before and after the sessions to evaluate perceptual changes after training. The results showed declines in color discrimination thresholds after training, as expected. Additionally, the training influenced outcomes across all three assessment types. After the training, the perceived color appearance changed near the trained color along the stimulus hue, and some of the unique hues and the color category boundaries moved significantly toward the trained color. These findings indicate that short-term repetitions of color discrimination training can alter color representations in the visual system, distorting color perception around the trained color.

## Introduction

Perceptual learning refers to the improvement of perceptual performance as a result of practice on perceptual tasks over several days. Previous studies have demonstrated that perceptual learning occurs for various types of perceptual tasks, such as luminance contrast detection^1^, orientation detection^2–4^, object recognition^5^, motion direction discrimination^6^, and stereopsis^7^. For instance, Sowden et al.^1^ trained observers to detect luminance contrast for ten days. The results showed that the average accuracy of each training day increased progressively, indicating that the training enhanced the perceptual performance for luminance contrast detection.

Color vision is also believed to be influenced by perceptual learning. For example, cherry farmers can rapidly and easily discriminate subtle differences in cherry color that are hard for ordinary people to perceive at a glance. One plausible explanation for this is that they have more exposure to cherries than ordinary people and thus undergo perceptual learning for cherry color. Although there are few studies on perceptual learning of color vision, some experiments suggest that perceptual learning takes place in basic color vision tasks such as color discrimination^8,9^.

Perceptual learning is known to exhibit stimulus specificity in certain conditions^10^, meaning that the learning effect only emerges under the same conditions as those trained. This specificity has also been observed in color vision perceptual learning. For instance, previous studies have shown that color discrimination performance improves only in the trained conditions after color discrimination training^8,9^. Grandison et al.^8^, for example, assigned observers to four groups with different combinations of trained hue (green and blue) and retinal location (upper and lower), and they conducted color discrimination training for eight days. The results revealed that the discrimination thresholds for the trained hue significantly decreased after training compared to before training, while no significant change was observed for the untrained hue or the untrained location. These studies indicate that perceptual training enhances color discrimination performance, but the effect is specific to the trained hue and retinal location. The retinal location specificity of the training effect suggests that color perceptual learning may take place at an early stage of visual processing with retinotopy. Likewise, Özgen and Davies^9^ also reported the specificity of color discrimination training to trained hues. They found that the perceptual learning effect did not transfer to untrained colors. This finding implies that color mechanisms representing specific hues may be involved in color perceptual learning.

In contrast, it is known that the effects of perceptual learning can sometimes transfer to tasks other than the ones trained depending on the tasks and stimuli employed. Many studies have shown that presenting task-irrelevant stimuli during training helps the transfer of the training effect to the location of the irrelevant stimuli or their judgments^11^. In addition, Chen and Fang^4^ trained observers on an orientation discrimination task for six days without using task-irrelevant stimuli or tasks. They showed that the observers improved their orientation discrimination ability and also shifted their perceived vertical direction toward the trained orientation. Likewise, McGovern et al.^12^ examined whether the training effect transferred between shape-related tasks of different complexity: orientation discrimination, curvature discrimination, and global form, using similar stimuli composed of multiple oriented elements. They assigned different combinations of these three tasks to the training and test phases. They reported significant transfer of the training effects across these different tasks. These transfers of the learning effects among the shape-related tasks suggest that perceptual learning in simple tasks may affect other perceptual tasks.

The transfer effects of color vision perceptual learning to different stimuli or tasks have not been well documented. Color perception encompasses various tasks that rely on different mechanisms, such as color difference perception and categorical color perception. For instance, color vision is considered to have a categorical nature: humans recognize the continuous spectrum of color as discrete categories such as “red,” “orange,” and “yellow”^13^. This feature of color vision is referred to as categorical color perception or color category. Previous studies have indicated a link between categorical color perception and color discrimination, a phenomenon called “category effect”^14–18^, though the interpretation of the underlying mechanisms is still controversial^19,20^. These studies reported that discriminating colors that belong to different color categories, such as green and blue, was easier and quicker than colors that belong to the same color category, even when the color difference was equal. In addition, training in color categorization has been reported to affect color discrimination performance. For instance, Grandison et al.^8^ trained observers to split green into two new categories for three days and observed a reduction in their green discrimination threshold compared to a control group that received no training. This effect was specific to the green region and did not generalize to the blue region, where no training was given. Similar results have been reported by other studies as well^9,21,22^. Based on these findings, it is conceivable that color discrimination training might also influence other color perception tasks, such as color category perception. However, this hypothesis has not been directly tested.

This study investigates how color discrimination training transfers to various aspects of color perception. Specifically, we assess whether color discrimination training affects categorical color perception, unique hues, and color appearance. The transfer effects, if any, will shed light on the underlying mechanisms of color vision plasticity.

## Experiment 1: The effects of color discrimination on various color perception

**Figure 1:**
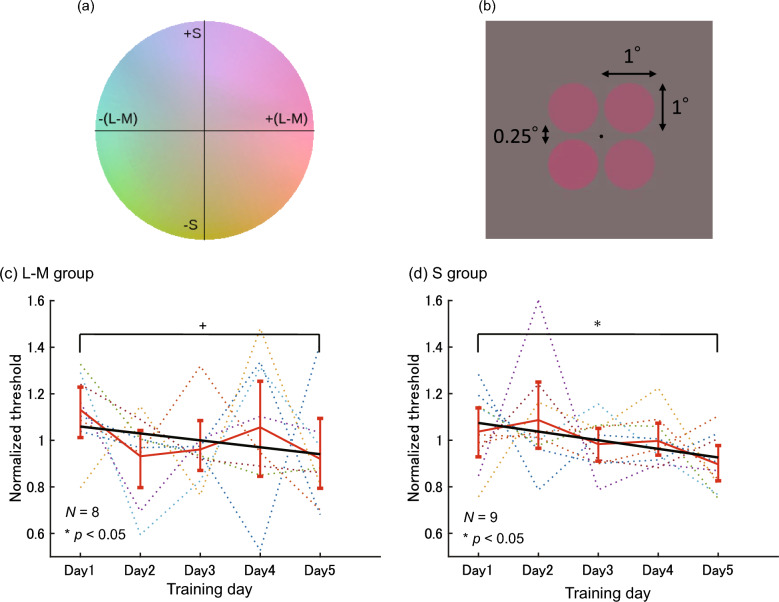
(**a**) MacLeod-Boynton chromaticity diagram. (**b**) Stimulus layout. (**c**,**d**) Normalized discrimination threshold for (**a**) L-M group and (**b**) S group in color discrimination training of Experiment 1. The horizontal axis represents the number of training days, and the vertical axis represents the normalized discrimination threshold. The dotted lines represent the results for each observer. The solid red line represents the mean of all observers for each day, and the error bars represent 95% confidence intervals from the bootstrap procedure with 10,000 repetitions. The solid black line shows the linear regression line fitted to the mean of all observers.

This series of experiments explores how training in color discrimination at a specific color affects other aspects of color perception. First, we ask the observers to perform a five-day training of color discrimination along the + L-M or + S direction at the 0° color on the MacLeod-Boynton chromaticity diagram^23^ (Fig. 1a, the color on the L-M axis in the positive direction). Then, we evaluate how training influences color categories and color appearance based on unique hues. Specifically, we conduct two types of tests before and after the training. Test 1 (a color category experiment) measures the category boundaries between orange-pink and pink-purple, which are the boundaries of basic color terms^24^ near the trained color. This test assesses whether the training effects affect the categorical color perception near the trained color. Test 2 (a unique hue experiment) measures the unique red near the trained color. This test evaluates whether the training effects affect the color appearance.

### Color discrimination training

Figure 1b shows the spatial arrangement of the experimental stimuli. The color of these stimuli is expressed using the hue angle $\theta$. Specifically, $\theta =0$ corresponds to the positive direction along the L-M axis, while $\theta =90$ corresponds to the positive direction along the S axis. The circles in the stimulus were colored with the 0° color except for one circle, which had a slightly different color in either the L-M or S positive direction from the 0° color. The observer identified this differently colored circle in a four-alternative forced choice manner. The color shift directions were assigned separately to two observer groups: the group that performed discrimination along the L-M axis was referred to as the L-M group (*N* = 8), and the group that performed discrimination along the S axis was called the S group (*N* = 9). Each observer performed 220 trials per day and completed five days of the experiment, leading to 1100 trials in total. Color difference adjustments were made using the PSI adaptive staircase method^25^, and the threshold for each day was estimated by fitting a psychometric function. The thresholds were divided by the mean threshold across the five days for each observer to focus on the trend during the training period. These values are referred to as normalized thresholds.

Figure 1c,d show the normalized thresholds for the L-M and S groups, respectively. For both groups, the normalized thresholds decreased over time, with some daily variations among observers within each group. The bootstrap test revealed that the slope of the regression line for the normalized thresholds was significantly negative for the S group (*p* < 0.05) and marginally significant for the L-M group (*p* < 0.10). These results indicate that the color discrimination training improved the color discrimination performance as expected.

The color discrimination threshold decreased significantly after training in the S group. The L-M group also showed a decreasing trend, although it was slightly below the significance level. We think that the insignificant threshold decrease in the L-M group might be caused by the small number of observers and trials, both of which could make the estimations of the mean discrimination thresholds less reliable. In particular, one observer had a high threshold on the fifth day. This observer also exhibited a monotonic decrease in threshold from the first day to the fourth day, suggesting a declining trend in color discrimination threshold. These results indicate that the five-day training successfully induced perceptual learning, which improved color discrimination performance. This trend is consistent with previous studies of perceptual learning for color search^22^ and color discrimination^8^. In the following sections, we examine whether the training effects of the color discrimination task transfer to other color perception tasks.

### Test 1: Color category boundary

Test 1 evaluated the color category boundaries before and after the color discrimination training. We measured the two boundaries around the trained color ($\theta = 0$): orange-pink and pink-purple. The experimental stimuli were similar to the ones for the discrimination training (Fig. 1b), except that all circles had the same color. For instance, when assessing orange-pink, the observer indicated whether the circles’ color appeared more orange or pink in a two-alternative forced choice manner. We adjusted the hue angle of the circle using the PSI adaptive staircase method. The hue angle that yielded a 50% response probability was estimated by fitting a psychometric function to the experimental data and defined as the category boundary.

**Figure 2:**
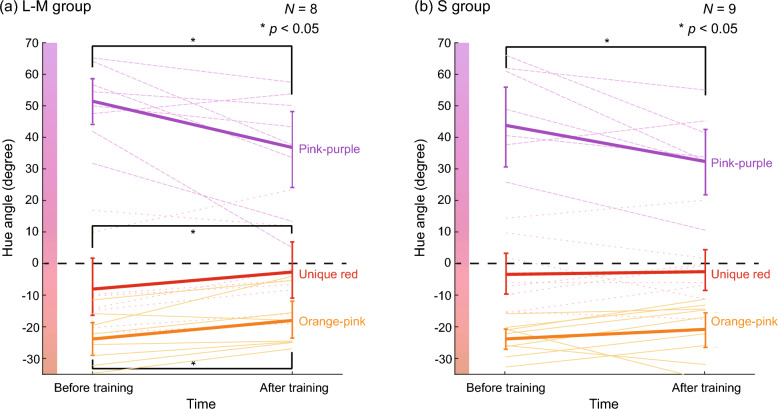
Color category boundaries and unique red measured in Test 1 and Test 2 of Experiment 1 for (**a**) L-M group and (**b**) S group. The horizontal axis shows before or after training, and the vertical axis shows the hue angle. The thin dotted lines represent the results for individual observers, and the bold lines represent the mean across all observers. The purple, red, and orange lines show the results of the pink-purple boundary, unique red, and orange-pink boundary, respectively. The black dotted line shows 0°, the trained color. Error bars indicate 95% confidence intervals obtained from the bootstrap procedure with 10,000 repetitions.

Figure 2 shows the color category boundaries before and after the training. The purple and orange lines in Fig. 2a represent the pink-purple and orange-pink boundaries for the L-M group, respectively. Both boundaries seemed to shift toward 0° after training. We tested the differences in category boundaries before and after training using the bootstrap procedure and found that the hue angle of the orange-pink boundary increased, and that of the pink-purple boundary decreased significantly (*p* < 0.05) after training. This indicates that the category boundaries shifted toward 0° after training, which was the training base color. The purple and orange lines in Fig. 2b show the pink-purple and orange-pink boundaries before and after the training for the S group, respectively. The trends seemed similar to those for the L-M group. The bootstrap test showed that there was no significant difference in the orange-pink boundary (*p* > 0.05), whereas there was a significant difference in the pink-purple boundary before and after training (*p* < 0.05). Supplementary Figure S1 shows the results of the pink-purple and orange-pink boundaries separately for the visibility of individual observers’ results.

In summary, for almost all the category boundaries and both observer groups, the color hues corresponding to category boundaries shifted toward 0°, which was used as the base color of the color discrimination training, after the color discrimination training. This suggests that the color discrimination training influenced judgments of color categories.

### Test 2: Unique hue

Test 2 assessed the unique red before and after training. The unique red is a hue that is perceptually neither yellowish nor bluish. We selected the unique red as the target because it is located near the training base color. The aim was to examine how the color discrimination training affected color appearance. The experimental method was almost the same as Test 1, except that the observer judged if the stimulus color appeared yellowish or bluish.

The red lines in Fig. 2a,b show the changes in the hue corresponding to unique red before and after the training for the L-M and S groups, respectively. In the L-M group, the unique red angle of all observers with negative values before training shifted toward 0° after training. In contrast, the unique red angle of observers with positive values before training changed inconsistently. We conducted a bootstrapping test to examine the statistical significance of the difference between before and after the training. The results indicated that the hue of unique red increased significantly after training (*p* < 0.05). In the S group, no consistent trend was found; the unique red hues of some observers shifted toward 0° after training, while those of others shifted in the opposite direction. A bootstrapping test at the 5% significance level showed that the change in unique red hue between before and after the training was not significant (*p* > 0.05). However, it should be noted that many (13 out of 17) observers in both groups exhibited shifts of the unique red hue towards 0°; the unique red hues increased after training for those who had negative hues before training, and decreased for the one who had a positive hue before training. Supplementary Figure S2 shows the results of the unique red separately from the category boundaries for the visibility of individual observers’ results.

### Discussion

In Experiment 1, we examined the effects of color discrimination training on different aspects of color perception. The training significantly reduced the discrimination threshold for the S group and marginally for the L-M group, indicating an increase in color discrimination performance. This is consistent with the results of previous studies on color discrimination training^8,9^ and thus not very surprising. In the category boundary test (Test 1), both groups shifted their pink-purple boundary significantly toward 0°, which was the trained hue. Additionally, for the L-M group, the orange-pink boundary also shifted toward 0°. Likewise, in Test 2, which measured the unique red, the unique red shifted significantly toward 0° in the L-M group, and many observers (13 out of 17) in both groups showed shifts of unique red toward 0° depending on the hue angle of unique red before the training. These findings imply that color discrimination training influenced color category judgments and unique hue.

How did the training affect the category and unique hue perception? For the L-M group, both pink-purple and orange-pink boundaries shifted toward 0°. A possible explanation is that repeated exposure to pink stimuli or repeating the color discrimination task during training narrowed their range of hues perceived as pink; in other words, the observers became more sensitive to pink and, therefore, could not perceive some hues as pink that they used to. For the S group, their pink-purple boundary changed significantly in a similar way as the L-M group; their perceptual color difference around the trained color seemed to expand. However, another interpretation is that the shift in the category boundary might have resulted not from the change of color representations due to the training but simply from the change in the color name labeling. The results of the unique red are somewhat helpful for discussing this possibility. As stated above, in the L-M group, the unique red also shifted toward the trained color 0°. Moreover, many observers, including the ones in the S group, showed individual shifts of unique red toward 0° depending on the hue angle of unique red before the training. These results support the idea that changes in color appearance (i.e., narrowing of hues seen as pink) altered their categorical color naming. Nevertheless, our results do not rule out the possibility of changes in color name labeling.

It is intriguing that both the L-M and S groups exhibited similar results. The shifts in the two category boundaries and the unique red were more pronounced along the S direction than along the L-M direction, as indicated by their small hue angles. Therefore, if color discrimination training were solely responsible for these shifts, one might expect stronger effects in the S group. However, the results did not reveal any such trend, though the differences between the L-M and S groups were not conclusive due to the small number of observers. This raises the question of whether color discrimination training itself played a crucial role in the observed shifts in color categories and unique hues. This issue will be revisited in the General Discussion.

Finally, the stimulus conditions in Experiment 1 were insufficient to understand the basic characteristics of the training effects. For instance, it did not directly test whether perceived color differences increased around the trained color or not. It also did not examine how the training affected other hues far from the trained hue. Furthermore, because we did not adopt the control observer group that skipped the training phase in Experiment 1, it remained unclear which of the training phase or the test phase induced the shift in category boundaries and unique hues. Hence, Experiment 2 investigated how color discrimination training changed their perception of color categories, unique hues, and color differences across the entire hues by employing two observer groups with different trained colors.

## Experiment 2: The effects of difference in trained colors

Experiment 2 examined how the differences in the trained colors affected color perception across the entire hues. The training involved five days of color discrimination in the + S direction at 0° or 180° (depending on observer groups, as described later) on the chromaticity plane. Three types of tests were conducted before and after the training: Test 1 – color difference perception, Test 2 – color category boundaries, and Test 3 – unique hue. Test 1 measured color difference perception at four hues: 0°, 90°, 180°, and 270°. The objective of Test 1 was to test the hypothesis that perceptual differences around the trained color increased after color discrimination training. Test 2 measured six color category boundaries in the entire hue circle: pink-purple, purple-blue, blue-green, green-yellow, yellow-orange, and orange-pink, which were the boundaries between basic color terms on the hue circle. Test 3 measured all unique hues: unique-red, unique-blue, unique-green, and unique-yellow. In each test, the perception changes due to color discrimination training were evaluated by comparing the pre- and post-training results.

### Training: Color discrimination

This section describes the five-day color discrimination training. The observers in different groups repeated a color discrimination task at their respective base colors: the 0° color (0° group, *N* = 10) and the 180° color (180° group, *N* = 10). The experimental procedure was similar to the training of Experiment 1. However, in Experiment 2, the observer used a laptop display for stimulus presentation and participated in the experiment at their own homes. Therefore, to ensure a robust training effect, we increased the number of trials per day to 320. As a result, the observer completed 1600 trials over the five-day experiment.

**Figure 3:**
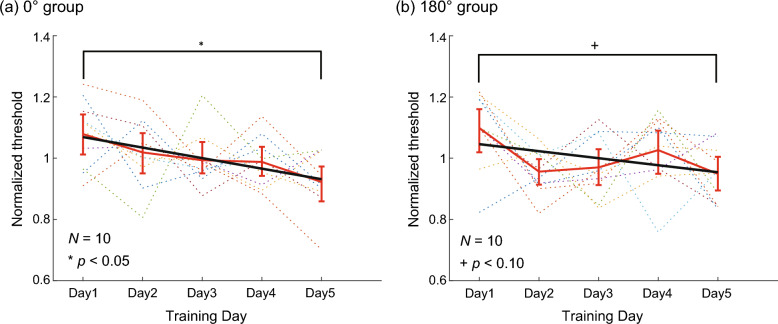
Color discrimination threshold in Experiment 2 for (**a**) 0° and (**b**) 180° groups. The format is the same as Fig. 1c,d.

Figure 3 shows the normalized color discrimination thresholds over five days for the 0° and 180° groups in (a) and (b), respectively. The thresholds varied daily for each observer, but the overall trend was a decrease in both groups. A bootstrap test confirmed that the training decreased the thresholds (and increased the performance) for the 0° group, as the slope of the regression line was significantly negative (*p* < 0.05). The slope was only marginally negative (*p* < 0.10) for the 180° group.

The five-day training significantly lowered the discrimination thresholds for the 0° group and marginally for the 180° group, indicating that perceptual learning occurred and enhanced color discrimination performance. This trend is consistent with Experiment 1 and previous studies on color perceptual learning^8^. We then measured different types of color perception before and after the training to examine whether the training influenced color perception across the entire hue circle.

The insignificance of the threshold decreasing trend for the 180° group might have resulted from several factors, as in Experiment 1. The first factor was the insufficient number of observers. Moreover, the uncontrolled experimental environment might have also increased the variability of the results. In particular, the visual stimuli might have changed depending on experimental environments. To check this issue, we measured the luminance and chromaticity of the display in a slightly bright room, which was covered by a curtain in the daytime, to examine the environmental effects. The results are summarized in Table S3 in Supplementary Materials. Compared with Table S2 (the measurement in the dark environment), the luminance was increased in the bright environment, while the chromaticity was barely affected. These unstable colors might have affected the color discrimination sensitivities, though we do not think their effects on the color changes by the environment were severe, considering that the environment equally affected different colors mainly along the luminance direction and that the color constancy or adaptation might have compensated for the color change^26^.

### Test 1: Color difference

**Figure 4:**
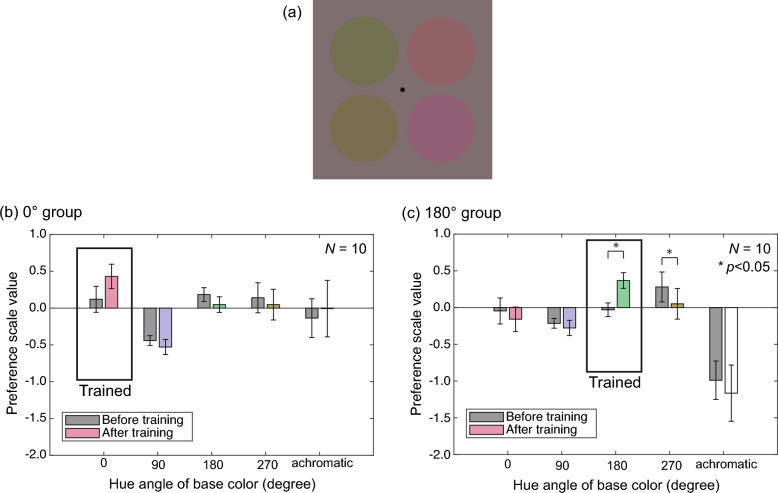
(**a**) Stimulus example in Test 1 of Experiment 2. The base colors on the left pair and right pair are 270° and 0°, respectively. (**b**,**c**) Preference scale values for (**b**) 0° and (**c**) 180° groups in color difference experiment. The horizontal axis shows the base color, and the vertical axis shows the preference scale value as an index of relative perceived color difference averaged across change levels. The gray and colored bars represent the results before and after training, respectively. The error bars are standard errors of mean across the observers.

This section describes Test 1, which measured the perception of suprathreshold color differences before and after the color discrimination training. This test aimed to investigate whether the training influenced color difference perception and, consequently, induced the shift of category boundaries found in Experiment 1. Figure 4a shows the stimuli used in this test. This stimulus consisted of four circles; two left and two right circles formed different color pairs, respectively. These color pairs were selected from two colors that shifted roughly along the hue direction from one of four base colors: 0°, 90°, 180°, or 270° colors. In addition, two achromatic colors that differ in luminance constituted a color pair. There were four levels of shift amounts (referred to as the “change level” hereafter) for each base color, resulting in 20 types of color pairs: 5 base colors × 4 change levels. Two of the 20 color pairs were presented on the left and right in a trial. We measured the perceptual color difference of every color pair using Thurstone’s paired comparison method.

Figure 4b,c show the preference scale values of the color difference for 0° and 180° groups, respectively. Because the change level monotonically increased the preference scale values, as shown in Supplementary Fig. S3, the scales were averaged across the change levels in Fig. 4b,c. In both groups, preference scale values did not largely differ between before and after the training. However, those only near the trained hue (0° and 180°, respectively) appear to be larger after training.

To examine the effects of the training group (0° or 180°), base color (0, 90, 180, or 270°), and time (before or after training) on preference scale values, we performed a three-way ANOVA. In this test, we conducted the Mendoza multisample sphericity test^27^ and applied Huynh–Feldt epsilon correction for violations of sphericity. The results are summarized in Table 1. Notably, the interaction between training group, color, and time was significant (*p* < 0.01). Posthoc tests revealed that, for the 180° group, there was a significant interaction between time and color (*F*(3.98, 35.8) = 7.47, *p* < 0.001), but only marginal for the 0° group (*F*(4, 36) = 1.86, *p* < 0.10). In addition, for 180° group, time had significant simple main effects at both 180° and 270° colors (*F*(1, 9) = 15.38, *p* < 0.01 for 180°, and *F*(1, 9) = 7.34, *p* < 0.05 for 270°).

**Table 1:** Results of three-way analysis of variance in perceptual color difference (Test 2).

Source	SS	df	MS	F-ratio	p-value
Group	2.04902	1	2.0492	5.6971	0.0282*
s × group	6.4744	18	0.3597		
Time	0.0019	1	0.0019	0.0557	0.8161 ns
Group × time	0.0434	1	0.0434	1.2480	0.2786 ns
s × group × time	0.6264	18	0.0348		
Color	18.2287	2.78	6.5606	5.6240	0.0027 **
Group × color	10.2508	2.78	3.6893	3.1626	0.0358 *
s × group × color	58.3427	50.01	1.1666		
Time × color	0.6288	3.48	0.1806	2.2014	0.0876 +
Group × time × color	1.3905	3.48	0.3993	4.8680	0.0027 **
s × group × time × color	5.1414	62.68	0.0820		
Total	103.1781	199	0.5185		

The interaction between time and color for the 180° group indicates that the relative sizes of perceived color differences across the hues changed before and after training. More specifically, perceived color differences increased at 180°, their trained color, and decreased at 270° after training. This is consistent with our hypothesis that the discrimination training would enlarge perceptual color differences near the trained color. However, for the 0° group, no significant changes in perceived color differences were observed after training, except for a slight increase at the trained color of 0°. Several factors might have caused the observed weak effect on perceived color difference. For instance, the results were highly variable across observers due to the difficulty of the supra-threshold color difference comparison task; this can be inferred from the large differences in the preference scale values of achromatic colors between 0° and 180° groups. The limited number of observers might have been insufficient to mitigate this substantial inter-observer variability. In addition, this problem might have been worsened by using colors with lower saturation, which might have made the judgment even harder. Consequently, the reliability of this color difference measurement should be regarded as low. In conclusion, there are only limited results suggesting that color discrimination training affects color difference perception.

### Test 2: Color category boundary

Test 2 measured perceptual color category boundaries before and after the training. We measured the six boundaries covering the entire hue: pink-purple, purple-blue, blue-green, green-yellow, yellow-orange, and orange-pink. The experimental methods were the same as Test 1 in Experiment 1 except for the number of measured color category boundaries.

**Figure 5:**
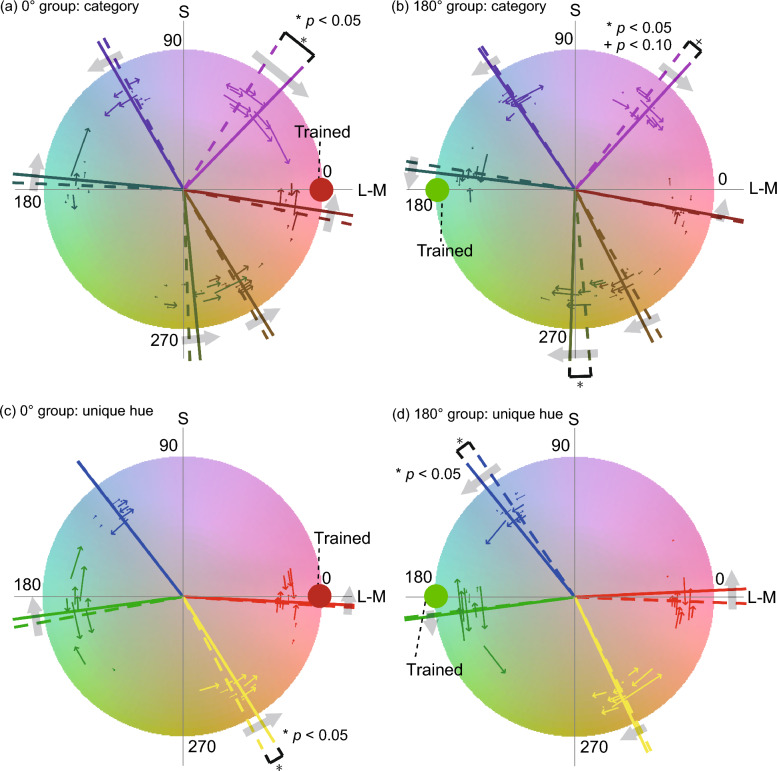
Category boundaries and unique hues on MB chromaticity diagram before and after training in Experiment 2. (**a**,**b**) Category boundaries for (**a**) 0° group and (**b**) 180° group. The dotted and solid radial lines show category boundaries before and after the training, respectively. The line colors indicate the kinds of boundaries (pink-purple, purple-blue, blue-green, green-yellow, yellow-orange, and orange-pink). The thin lines with arrows near the boundaries along roughly hue directions indicate the boundary shifts of individual observers. The gray arrows show the shift directions due to the training regardless of statistical significance. (**c**,**d**) Unique hues for (**c**) 0° and (**d**) 180° groups. The format is the same as (**a**,**b**).

Figure 5a,b illustrate the color category boundaries on the MB chromaticity diagram for the 0° and 180° groups, respectively. Some category boundaries seemed to shift considerably. In addition, there are some boundaries shifted toward the opposite directions between the training groups; for instance, the green-yellow and yellow-orange boundaries shifted toward the positive direction for the 0° group but toward the negative direction for the 180° group. In contrast, the pink-purple boundary shifted toward the negative direction after training for both groups. To assess the statistical significance of the boundary difference before and after the training, we conducted a bootstrap test at a 5% significance level with the Holm method^28^ to correct the significance level for multiple comparisons. The results indicated that in the 0° group, the pink-purple boundary showed a significant shift (*p* < 0.05), consistent with the results in Test 1 using the same trained color. In the 180° group, the green-yellow boundary showed a significant shift (p < 0.05), and the pink-purple boundary showed a marginally significant shift (*p* < 0.10).

Some color category boundaries of both groups shifted toward the trained color after training on a specific color, although not all categories shifted significantly. However, individual data also revealed interesting patterns. Some individual boundaries, such as the orange-pink boundary in the 0° group, shifted toward the trained color of 0° after training, even though the mean shift across observers was not significant. This lack of significance could be because the direction of the individual shifts depended on the initial position of the boundary before the training. Therefore, we performed another bootstrap test to examine whether the distance between the trained color and the category boundaries decreased after training, regardless of the shift direction. The results showed that in the 0° group, the pink-purple, blue-green, and orange-pink boundaries all shifted significantly toward the trained color. In particular, the orange-pink boundary replicated the results of Test 1 in Experiment 1. In the 180° group, the green-yellow boundary shifted significantly toward the trained color, and the blue-green boundary showed a marginal shift (*p* < 0.10). These results suggest that color discrimination training induces shifts of the color category boundaries toward the trained color.

### Test 3: Unique hue

This section describes Test 3, which measured unique hues before and after color discrimination training. We measured all of the four unique hues: unique red, unique blue, unique green, and unique yellow. The methods were the same as Test 2 in Experiment 1, except that the four unique hues, not just the unique red, were measured.

Figure 5c,d shows the changes in unique hues on the MB chromaticity diagram before and after the training. Figure 5c shows the results for the 0° group and Fig. 5d for the 180° group. Some unique hues seemed to shift after training in both groups. We assessed the statistical significance of the pre-post changes using a bootstrap test at a 5% significance level with the Holm method that corrected the significance level for multiple comparisons. The results revealed that in the 0° group, the unique yellow shifted significantly toward 0°, the trained color, after training. In the 180° group, the unique blue shifted significantly toward 180° after training.

The unique hues that moved significantly in both groups shifted toward the trained color after training. These results are similar to the finding that some color category boundaries shifted toward the trained color after training in Test 2. However, individual differences also exhibited interesting patterns like Test 2. For instance, the unique red in the 0° group, which showed a significant shift toward 0° in Experiment 1, did not show a significant shift. The reason for this absence of shift may be the individual differences in unique red before the training: below or above 0°. The observers with the unique red below and above 0° exhibited positive and negative shifts after training, respectively; in other words, the distances between 0° and the unique hue decreased after the training in most observers, leading to the cancelation of the shifts among observers. We performed a bootstrap test with 10,000 repetitions and a significance level of 0.05 to verify the statistical significance of this trend, which was whether the difference between the trained color and the unique hue decreased after training, regardless of the shift direction. The results showed that the unique red and unique yellow in the 0° group and the unique blue in the 180° group significantly approached the trained color after training. All these results with similar trends, along with the partial increase in perceived color difference around the trained colors in Test 1, raise the possibility that color discrimination training altered color appearance toward the trained color, resulting in shifts of color category boundaries and unique hues in the same direction. The following section will further discuss this possibility by comparing the shifts of color category boundaries and unique hues.

### Comparison between Tests 2 and 3

Tests 2 and 3 measured the shifts of color category boundaries and unique hues after training and found similar patterns around the trained colors. This implies that the same factors might have affected the shifts in color category boundaries and unique hues. Therefore, we conducted a combined analysis of the results of Tests 2 and 3 to examine the common characteristics of changes in color category boundaries and unique hues across training groups.

**Figure 6:**
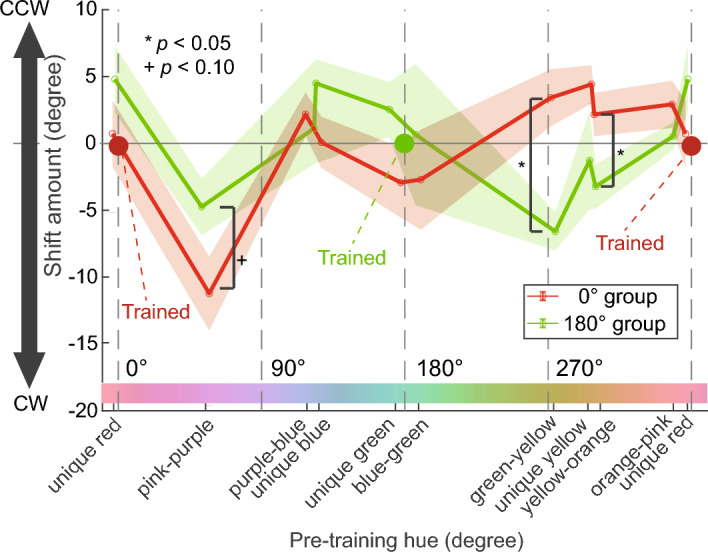
Shift amounts of categories (color category boundaries and unique hues in Tests 2 and 3) before and after training in Experiment 2. The horizontal axis represents the pre-training hue angle, and the vertical axis represents the category shift amount; positive and negative values indicate that the category shifted counterclockwise and clockwise due to training, respectively. The red and green lines show the results of the 0°and 180° groups, respectively. The error zones (the shaded areas) show the standard errors of mean across observers.

First, we computed the shift amount of each category boundary and unique hue before and after training by subtracting the pre-training hue angle from the post-training hue angle. For simplicity, we refer to each category boundary and each unique hue as just a “category” in this section. Figure 6 shows the category shift amounts. Positive and negative values indicate that the category shifted counterclockwise and clockwise after training, respectively. In this figure, the category shift direction seems roughly symmetrical to the zero-shift line (the black horizontal line) between the training groups for all categories except the pink-purple boundary. To verify this tendency, we performed a two-way ANOVA (training group × category) on the category shift amounts. The results showed that the main effect of the category was significant (*F*(9, 162) = 2.74, *p* < 0.01), and the interaction between the training group and category was also significant (*F*(9, 162) = 2.23, *p* < 0.05). According to the posthoc test, the simple main effect of the training group was significant for green-yellow and yellow-orange categories (*F*(1, 18) = 13.76, *p* < 0.01 for green-yellow, and *F*(1, 18) = 4.83,* p* < 0.05 for yellow-orange), and marginally significant for pink-purple category (*F*(1, 18) = 3.16, *p* < 0.10). These results indicate that the shift direction tends to be almost symmetrical between the training groups. Moreover, in Fig. 6, the shift amounts seemed to change from positive to negative at the trained color (0° or 180°) for both the 0° and 180° groups. This tendency means that the categories shifted toward the trained color. In other words, this result is in line with our previous findings that the categories near the trained color tend to move closer to the trained color after the training.

### Discussion

In Experiment 2, we assessed the color difference, category boundaries, and unique hues across various hues for two groups of observers who repeated color discrimination at 0° or 180°, respectively. The results showed that, as in Experiment 1, the category boundaries and the unique hues shifted towards the trained colors. Moreover, the directions of the shifts were approximately reversed between the groups. Because the test tasks were identical between the observer groups, the opposite shifts between the observer groups should have resulted from the color discrimination training, not from the repetition of the test tasks. These results suggest that the repetition of color discrimination altered the category boundaries and unique hue, especially around the trained color. In addition, the category and unique hue shifted in a similar direction, suggesting that the same factor influenced these shifts.

However, some of the experimental results were unstable. The improvement of the color discrimination performance was only marginally significant in the 180° group. In addition, the results of the color difference perception were not very informative due to some factors: the difficulty of the task for observers, the limited number of participants, the observation of significant changes exclusively in the 0° group, and the restriction to only four hues in measurement. To address these limitations, we additionally conducted Experiment 3, which assessed the color appearance of the entire hue using a modified elementary color naming.

## Experiment 3: The change in color appearance in the entire hue

In Experiment 3, we replaced the color difference test, which yielded unclear results in Experiment 2, with a test that measured the color appearance of the entire hue circle using a modified elementary color naming before and after the color discrimination training. We also asked the observers to perform a categorical color naming task to verify the shift in the category boundary observed in the previous experiments. Furthermore, to enhance test precision, the observers conducted the test tasks in a dark room rather than in their own homes.

### Training: Color discrimination

**Figure 7:**
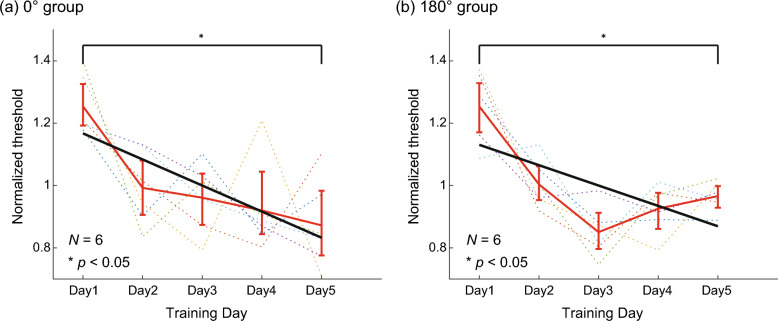
Color discrimination threshold in Experiment 3 for (**a**) 0° and (**b**) 180° groups. The format is the same as Fig. 1c,d.

We used the same method for the color discrimination training as in Experiment 2. We recruited six observers in each of the 0° and 180° groups. Figure 7a,b show the results of the 0° and 180° groups, respectively. Both groups showed improved performance with repeated color discrimination; the slopes of the linear regression lines were significantly negative for both groups.

### Test 1 and 2: Elementary color naming and categorical color naming

The observer performed the test tasks on the days before and after the color discrimination training. The stimuli had the same layout as in test 2 (category boundary) of Experiment 2, while the test tasks were conducted in a dark room of our laboratory. In each trial, one of the 36 colors chosen from the hue circle at 10° steps was shown as the color of four circles. The observer reported the appearance of this hue in a two-step procedure akin to elementary color naming. In the first step, the observer selected two hues from the basic four hues (red, blue, green, and yellow) that constituted the color appearance using mouse clicks. In the second step, a dark-gray scale bar appeared below the stimulus, and then the observer indicated the proportion of the two hues in the color appearance using the visual analog scale on the scale bar. After that, the observer named the stimulus color using categorical color naming; specifically, they used the numeric keypad to pick the color name that best described the color appearance from the six basic color terms of Berlin and Kay^24^ (green, yellow, blue, purple, pink, and orange). The observer repeated this trial three times for each of the 36 colors and completed all the trials in one session.

**Figure 8:**
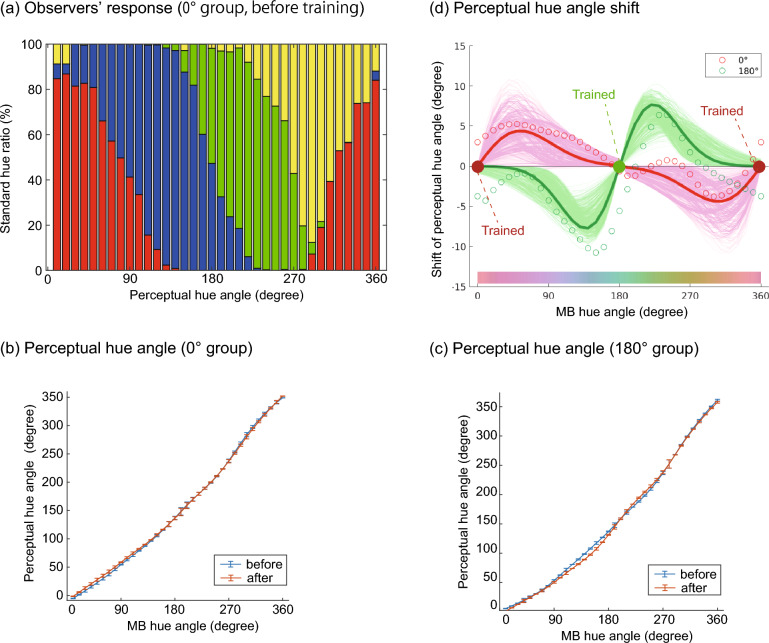
Results of elementary color naming in Experiment 3. (**a**) The proportion of the opponent color responses (an example from the 0° group before training). (**b**,**c**) The comparison of the hue angle in the perceptual opponent color space before and after training for 0° and 180° groups, respectively. The horizontal and vertical axes show the hue angles in the MB chromaticity diagram and in the perceptual opponent color space, respectively. The line colors indicate the before- and after-training conditions. (**d**) The change in the perceptual hue angle before- and after-training as a function of the MB hue angle. The horizontal axis shows the hue angle in the MB chromaticity diagram, and the vertical axis shows the change in the perceptual hue angle. A positive value on the vertical axis means that the perceptual hue angle shifted counterclockwise after training (as in Fig. 6). The line colors show the training groups. The circle plots show the hue shift measured in the experiment, the bold lines show the fitted derivative Gaussian, and the thin lines show the bootstrap samples of the fitted derivative Gaussian.

The elementary color naming results were analyzed as follows. First, the responses of all observers were summed up, and the response ratios were calculated for each color, as shown in Fig. 8a. Then, the hue angle of each color in the perceptual opponent color space (0°, 90°, 180°, and 270° correspond to red, blue, green, and yellow) from the vector sum of the four color components in the observer responses. The hue angles in the perceptual opponent space as a function of the MB hue angle for the 0° and 180° groups are shown in Fig. 8b,c, respectively. Finally, the difference in the perceptual hue angle before and after the color discrimination training was computed. Figure 8d shows the perceptual hue shifts for both observer groups. A positive value on the vertical axis indicates that the perceptual hue angle increased after the training. In this figure, the perceptual hue angle tended to increase when the hue angle exceeded the trained color (0° or 180°) and to decrease when the hue angle fell below the training color. This indicates that the differences in the perceptual hue around the trained color were expanded after the training. A derivative Gaussian function centered on the trained color, which resembles the pattern of the experimental results, was fitted to the hue angle shift with a nonlinear least squares regression to capture this tendency quantitatively. The free parameters were the amplitude and width of the derivative Gaussian, and a nonparametric bootstrap test with 10,000 repetitions was used to test whether the amplitude was negative (A negative coefficient means that the perceptual hue angle shifted towards the negative (positive) direction when the MB hue angle was lower (higher) than the trained color). The results showed that the amplitude was significantly negative (*p* < 0.05) in both groups. This indicates that the differences in color appearance around the trained color expanded after the discrimination training. In addition, even when we used the center of the derivative Gaussian as an additional free parameter, the results were slightly unstable in the 0° group, but the 95% confidence interval of the derivative Gaussian center was 176.3°–212.6° in the 180° group, which was close to the trained color. This is also consistent with the expansion of the perceived color near the training color. This result is shown in Fig. S4 in the Supplementary Materials.

**Figure 9:**
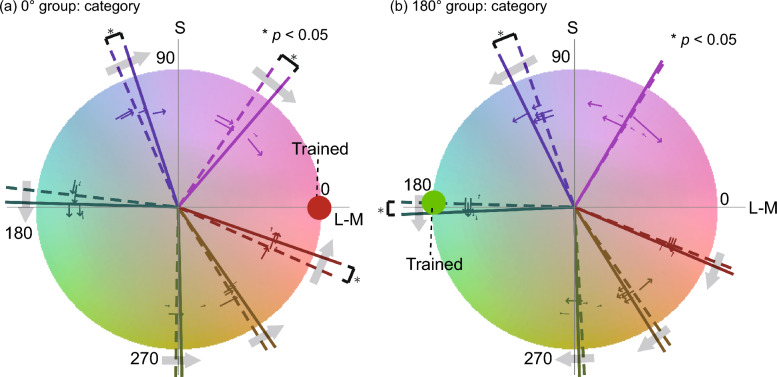
Category boundaries on MB chromaticity diagram before and after training in Experiment 3. (**a**,**b**) Category boundaries for (**a**) 0° group and (**b**) 180° group. The format is the same as Fig. 5a,b.

The categorical color naming results were analyzed as follows. First, we assigned one of the basic color terms that received the highest response rate from all observers to each of the 36 colors. Then, we determined the category boundary based on the assigned color categories. Figure 9a,b show the category boundaries in the same format as Fig. 5a. We assessed the statistical significance of the category boundary shift using the nonparametric bootstrap method after adjusting the significance level with the Holm method. The results showed that, despite the different experimental methods, some of the category boundaries shifted significantly towards the trained color, consistent with the shift of category boundaries measured in Experiments 1 and 2.

These results corroborated our previous findings. First, the differences in color appearance were expanded around the trained color after the color discrimination training. Moreover, the category boundaries also shifted towards the trained color as well. Furthermore, as in Experiment 2, the patterns of the color appearance and category shift of the 0° group and the 180° group were approximately inverted, indicating that their shifts were a consequence of the color discrimination training rather than mere repetition of test tasks.

## General discussion

This study examined how color discrimination training affects color categories and color perception. In experiment 1, we measured the changes in the color category boundaries (orange-pink and pink-purple) and unique red around the trained color before and after training, with the trained color set at 0° on the MB chromaticity diagram. In the results, the discrimination threshold decreased, indicating a clear perceptual learning effect. More importantly, color categories and unique red moved significantly toward 0°, the trained color, in most conditions after the training. Experiment 2 assessed the impact of color discrimination training on the whole hue circle and also investigated whether the training effect on color category boundaries and unique hues was caused by the change in perceptual color difference. We assigned the observers to groups that trained color discrimination at 0° or 180° and measured the color category boundaries and unique hue on the entire hue circle, as well as the perceived color difference. The results revealed that, in the 180° group, the perceived color difference increased significantly near the trained color. Furthermore, color category boundaries and unique hues shifted significantly after color discrimination training. Most importantly, the shift of the color category boundaries and unique hues near the trained color was toward the trained color. Finally, Experiment 3 assessed the change in color appearance on many colors from the entire hue circle due to color discrimination training. In the results, the differences in perceived hue were expanded near the trained color (0° or 180°) after the training. These results suggest that five days of color discrimination training alters the color category boundaries, unique hues, and color appearance. One possible explanation is that the expansion of the perceptual color difference near the trained color is attributed to these effects of the training.

This study has important implications for showing that color discrimination training has a broad influence on various aspects of color perception. Previous studies have reported perceptual learning on color discrimination^8,9^ and often linked it to the so-called category effect, where color discrimination performance improves at color category boundaries. Most of them have indicated that color discrimination performance increases with training, and the training effect is confined to the trained color. These results raise the possibility that the category effect may be acquired through repeating color judgment near the category boundaries. On the other hand, it has also been shown that, after repeatedly performing the color grouping task, color discrimination performance was enhanced at colors that matched the trained color group boundary^22^. These studies referred to the connection between the training and the category effect but mainly discussed the results based on the framework of color discrimination. In contrast, the tests conducted in this study measured color appearance and color categories, whose mechanisms are considered greatly different from those of color discrimination^29,30^. Although this study cannot discuss the association between the category effect and color perceptual learning, it offers insights into color perceptual learning that are distinct from those discussed in previous studies; more specifically, it demonstrated the effect of color discrimination training for just a few days on general color perception, such as the changes in color appearance and color category boundaries.

How did color discrimination training alter the color representation in the visual system? Although we cannot provide a definitive answer to this question based only on our experimental results, our results have some clues. One of them is that the results of different tasks, such as color appearance, color category boundary, and unique hue, exhibited similar patterns near the trained color. First, the changes in color appearance along hues near the trained color were increased after the training in Experiment 3. Also, color categories and unique hues near the trained color shifted toward the trained color after training in many conditions. These results are consistent with the idea that the perceptual color difference near the trained color increased due to training and consequently influenced all three types of tasks similarly. However, there were some shifts in category boundaries and unique hues that were difficult to interpret; for instance, the pink-purple boundary shifted in the same directions in both 0° and 180° groups in Experiment 2. These unstable aspects of the results make the conclusions rather unclear. The main reason for this is considered to be the large individual variability in the experimental results, the small number of observers, and the limited number of trials allocated to our test trials to prevent unexpected training effects from the test tasks. The experimental accuracy could be improved by increasing the number of observers.

The causal relationship between color discrimination training and the changes in other color-related tasks remains unclear. The color category boundaries and unique hues might have shifted not because of the task repetition but because of the mere exposure to the color discrimination stimuli. This hypothesis is consistent with the long-term color adaptation phenomenon reported by some previous studies^31–33^. Neitz et al.^31^ showed that unique yellow changed after the observers wore colored contact lenses for hundreds of hours. This suggests that the color appearance is affected by the long exposure to unbalanced colored visual input rather than by the task repetition. Furthermore, the improvement in color discrimination thresholds due to training in the current study was significant but small, which questions the necessity of the color discrimination task for the shifts in the color category boundaries and unique hues. However, our results were obtained in a much shorter training period, only a few tens of minutes in total, as compared with the experiment by Neiz et al. Although Belmore and Shevell^34,35^ also reported a similar shift of unique hue after exposure to red stimuli on a CRT display for a shorter period (1 h/day × 12 or 14 days), the exposure time was still much longer than our experiments. Therefore, it is possible that performing the task directly or indirectly induced stronger effects on color perception change after training. Unfortunately, our results cannot distinguish between these two possibilities: task repetition or exposure. Further experiments are needed, for example, by dividing the observers into two groups, one of which performs the task, and the other does not.

This study showed that various aspects of color perception changed after color discrimination training, but the underlying mechanisms and the basic characteristics of this phenomenon are still unknown. As we mentioned above, we do not know if the color discrimination task or the mere exposure to color stimuli caused the shift in color category boundaries after training. We also did not investigate the temporal characteristics, such as the duration of the training period required to induce the category boundary shift and the persistence of the shift after the training. The color range, including the luminance dimension, affected by the color discrimination training has not been widely investigated in the current study. Moreover, we could only superficially discuss what level of color representations changed due to training. Because all of these characteristics are essential to understanding the mechanism more deeply, future studies should address these issues. However, the current study revealed that a short period of color discrimination training could alter the wide-ranging characteristics of color perception, suggesting that such training might change color representations in the visual system that affect different aspects of color vision. This is an important step in understanding the relationship between color perception and our daily experiences.

## Methods

### Experiment 1: General methods

#### Overall flow of experiment

We used the “test-training-test” paradigm, a standard perceptual learning paradigm, to examine the effects of color discrimination training. This paradigm compares the performance of the same test task before and after the training to assess how the training influences perceptual performance for the test.

Table 2 summarizes the overall experimental flow for each observer in Experiment 1. The training lasted for five days, during which the observers repeated the color discrimination task with the same color. The test was conducted one day before and one day after the training. The observers performed two test tasks each day, and the order of the two tests was counterbalanced across observers to avoid any order effects. Thus, the total duration of the experiment was seven days, but we allowed a maximum gap of two days between the experiment days.

**Table 2:** Flow of Experiment 1.

Day1	Day2 ~ Day6	Day7
Test 1: Color category boundary	Training: Color discrimination	Test 1: Color category boundary
Test 2: Unique hue		Test 2: Unique hue

#### Observers

Seventeen undergraduate and graduate students and faculty members (4 female, aged 22–57 years) from Tokyo Institute of Technology participated in the experiment. Four of them had previous experience with a similar color perception learning experiment in the same laboratory. All observers had normal or corrected-to-normal visual acuity and passed the Ishihara Color Vision Scale II. Based on the color directions to be discriminated in the training, we divided the observers into two groups: the L-M group (8 observers) and the S group (9 observers). We called this difference the “training group.” To enhance the training effect, we assigned the observers with prior experience in the previous experiment to different training groups.

#### Ethical approval and informed consent

The Ethical Review Committee of Tokyo Institute of Technology approved all the experiments in this study, which followed the Declaration of Helsinki. All observers gave written informed consent.

#### Apparatus

The experiment was conducted in a darkroom with a blackout curtain. Stimuli were presented on a liquid crystal display (LCD: ColorEdge CG2730, EIZO, Japan) placed in the darkroom. The display was connected to a computer (Pavilion, HP Japan, Japan: GeForce RTX 2060 Rev. A and Ubuntu 18.04) and had a resolution of 2560 × 1440 pixels and a refresh rate of 59.95 Hz. A colorimeter (ColorCAL II, Cambridge Research Systems, UK) was used to measure the gamma characteristics of the red, green, and blue primaries, and a spectroradiometer (Specbos 1211–2, JETI, Germany) was used to measure the spectral distributions of the primaries and that of black. The display was carefully calibrated using the measured photometrical values to present the desired levels of luminance and chromaticity accurately. Table S1 in Supplementary Materials summarizes the intended and measured luminance and chromaticity (*u’* and *v’*) for several stimulus colors, as determined using the spectroradiometer.

Programs written by the authors on MATLAB R2020b and Psychtoolbox 3^36,37^ controlled the experiment, including stimulus presentation and observer response collection. The observers used a numeric keypad to respond during the experiment. The observers viewed the stimuli from a distance of 75 cm in the darkroom, with their heads approximately positioned on a chin rest.

#### Stimulus: color space

We used a modified version of the MacLeod-Boynton (MB) chromaticity diagram^23^, shown in Fig. 1a, to define the stimulus colors. This chromaticity diagram consists of the L-M and S axes based on the cone spectral sensitivity proposed by Stockman and Sharpe^38^. The origin was metameric to the equal energy white. The L-M and S axes were normalized according to the color gamut of the monitor; the maximum absolute values on each axis were defined as a unit distance.

The stimulus colors are represented with two symbols: $s$ and $\theta$. $s$ is saturation, the distance from the origin in the MB chromaticity diagram. $\theta$ is hue angle; $\theta =0$ corresponds to the positive direction on the L-M axis, and $\theta =90$ corresponds to the positive direction on the S axis. The stimulus luminance was fixed at 20 cd/m^2^ unless other values were specified in this paper.

#### Stimulus: spatial layout

Figure 1b shows the spatial layout of the stimulus, which was the same for all color discrimination training, Tests 1 and 2. The background color was the color of the origin of the chromaticity plane at 20 cd/m^2^ in all the experiments, and a fixation point and four circles appeared on the background. Each circle had a size of 1° × 1° in visual angle, and the edge of each circle was 0.25° away from that of the adjacent circle. The colors of these circles were set differently for training and each test, as described in detail in the following sections.

### Experiment 1 Training: Color discrimination training

This section describes the methods and results of the color discrimination training that was conducted over five days. The observers were divided into two groups, L-M and S, based on hue angles to be discriminated. The aim of this experiment is to induce an increase in color discrimination sensitivities through the five-day training.

#### Stimulus

Figure 1b illustrates an example of the stimulus. It consisted of four circles, only one of which had a different color. The three circles with the same color are referred to as the “reference circles,” and the stimulus with a different color as the “target circle.” The color of the reference and test circles are referred to as the “reference color” and “target color,” respectively. A black fixation point was located at the center of the stimulus.

The reference color had a chromaticity of $(\theta , s) = (0, 0.81)$. The target color varied from the reference color along the L-M positive direction for the L-M group and along the S positive direction for the S group. Thus, the L-M group and S group discriminated color changes mainly in saturation and hue directions, respectively. The color difference between the reference and target colors was controlled during the experiment based on the observer’s responses with the PSI adaptive staircase method.

#### Procedure

A stimulus was presented for 0.5 s in each trial, followed by a gray background with only a fixation point. The observer was requested to fixate at the fixation point and had to indicate which of the four circles had a different color from the others in a four-alternative forced choice (4AFC) manner. The observer pressed a key corresponding to the position of the circle: ‘4’ for upper left, ‘5’ for upper right, ‘1’ for lower left, and ‘2’ for lower right. An auditory feedback sounded for incorrect responses. After the response, the background was presented for 0.3 s before the next trial. We employed this stimulus duration considering the previous suggestion that color discrimination sensitivities get stable in stimulus durations longer than 500 ms^39,40^.

Each session consisted of 220 trials. The first 20 trials were practice trials to help the observers adapt to the stimuli and familiarize themselves with the task. We did not analyze the responses from the practice trials. The observer completed one session per day. The sessions were as consecutive as possible over five days, with a maximum gap of two days. We obtained 1100 responses in total, 1000 of which were analyzed.

#### Analysis

We fitted a logistic function to the correct and incorrect responses as a function of color differences using a maximum likelihood criterion. The Palamedes Toolbox^41^ was utilized for the regression. The discrimination threshold was defined as the color difference at the inflection point of the logistic function, which corresponded to a correct response ratio of 0.615; this ratio was the midpoint between the chance level of 0.25 and the upper asymptote of the logistic function 0.98, accounting for the response errors. Additionally, a bootstrap procedure with 10,000 resamples based on the fitted logistic function was performed to obtain 95% confidence intervals for the thresholds. This bootstrap procedure integrated both the parametric and nonparametric bootstrap. First, we used a parametric bootstrap procedure, which assumed that the correct response rate of each observer followed a logistic function to assess intra-observer variability. Then, we applied a nonparametric bootstrap procedure to those parametric bootstrap samples to assess inter-observer variability; in this procedure, we randomly sampled the observers with replacement, and their parametric bootstrap samples were used to assess the data variability. The bootstrap method for experimental results analyzed with psychometric functions in this study followed this procedure.

Thresholds were estimated for each observer and day. Because the mean thresholds across the five days differed greatly among observers, we normalized the thresholds for each day by dividing them by the mean threshold across the five days. The obtained values were referred to as normalized thresholds. Finally, a linear regression was performed for the normalized thresholds based on the least-squares criterion to see the threshold trend across the training days. We statistically tested whether the slope of the regression line was negative using the bootstrap procedure with a significance level of 0.05.

### Experiment 1 Test 1: Color category boundary

#### Stimulus

The stimulus consisted of four circles with the same color. We adjusted the hue $\theta$ of the circle color using the PSI adaptive staircase method based on the observer’s response. The saturation was fixed at $s=0.81$.

#### Procedure

The orange-pink and pink-purple boundaries were measured in separate sessions, and the order of the two conditions was counterbalanced across observers to avoid any order bias. Each trial started with a stimulus presentation for 0.5 s, followed by a gray background with a fixation point. The observer had to judge the color of the four circles in a two-alternative forced choice (2AFC) manner. In the orange-pink session, they pressed ‘6’ if the color was pink or ‘4’ if it was not pink (i.e., orange). In the pink-purple session, they pressed ‘6’ if the color was purple or ‘4’ if it was not purple (i.e., pink). Then, five colors were randomly selected from a whole hue circle with $s=0.81$ and presented for 0.1 s each to prevent adaptation to the stimulus color. The next trial began 0.3 s after the response, and the stimulus appeared again. Each session had 100 trials. The observer conducted one session for each boundary.

#### Analysis

We used a psychometric function to analyze the data obtained from each observer. First, we coded each response as 0 or 1; for example, for the orange-pink boundary, we assigned 1 to ‘pink’ responses and 0 to ‘not pink’ responses. We then calculated the response ratios for each hue angle and fitted a logistic function as a psychometric function using the Palamedes Toolbox. Finally, we defined a category boundary as a hue angle corresponding to a response ratio of 0.5. In addition, we performed the bootstrap procedure with 10,000 repetitions based on the fitted logistic function to obtain 95% confidence intervals for the category boundaries.

### Experiment 1 Test 2: Unique hue

The stimuli for Test 2 were the same as those for Test 1. The procedure was also similar to Test 1, except for the judgment criterion. The observer had to judge whether the four circles were more bluish or more yellowish in a 2AFC manner by pressing the key ‘4’ for yellowish and ‘6’ for bluish. Finally, as in Test 1, we coded the responses as 1 for bluish and 0 for yellowish and then fitted the psychometric function.

### Experiment 2: General methods

#### Overall flow of experiment

The experiment followed the test-training-test paradigm as in Experiment 1. Table 3 summarizes the overall experimental procedure for each observer in Experiment 2. The schedule was the same as in Experiment 1, except for the experimental equipment and some experimental procedures, as described later.

**Table 3:** Flow of Experiment 2.

Day1	Day2 ~ Day6	Day7
Test 1: Color difference	Training: Color discrimination	Test 1: Color difference
Test 2: Color category boundary		Test 2: Color category boundary
Test 3: Unique hue		Test 3: Unique hue

#### Observers

A total of 20 Tokyo Tech undergraduate and graduate students, faculty members, and an external observer took part in the experiment as observers (6 females, age range 21–43 years). Two of them had also participated in Experiment 1. All observers had normal or corrected-to-normal visual acuity and passed the Ishihara color test.

The observers were divided into two groups based on the base color of color discrimination training: 0° group (10 observers) and 180° group (10 observers). The base colors of the color discrimination training were 0° and 180° for 0° and 180° groups, respectively. We call these groups the training color groups. Two observers who had previously participated in Experiment 1 were assigned to the 0° and 180° groups, one in each group.

#### Ethical approval and informed consent

The Ethical Review Committee of Tokyo Institute of Technology approved all the experiments in this study, which followed the Declaration of Helsinki. All observers gave written informed consent.

#### Apparatus

The experiment was conducted at the observers’ homes for convenience. Stimuli were presented on the non-glare LCD of a loaned laptop PC (ProBook, HP Japan, Japan: Ubuntu 20.04 and Intel Core i5-1135G7, Intel Xe Graphics TGL GT2). The display had a resolution of 1920 × 1080 pixels and a refresh rate of 60.01 Hz. A colorimeter (ColorCAL II, Cambridge Research Systems, UK) was used to measure the gamma characteristics of the display, and a spectroradiometer (Specbos 1211–2, JETI, Germany) was used to measure the spectral distributions of red, green, and blue primaries and that of black. The measured photometric values were used to accurately present the desired levels of luminance and chromaticity. Table S2 in Supplementary Materials summarizes the intended and measured luminance and chromaticity (*u’* and *v’*) for several stimulus colors, as determined using the spectroradiometer.

A program written by the authors on MATLAB R2021a and Psychtoolbox3 controlled the experiment. During the experiment, observers responded using the keyboard of the laptop. The observers viewed the stimuli from a distance of approximately 50 cm without a chin rest. They were instructed to keep the experimental environment as consistent and dark as possible throughout the experimental period.

#### Stimulus

The color space and spatial layout of the stimulus were the same as in Experiment 1 (see Fig. 1b). The colors of the four circles differed across the training and tests, as described in the following sections.

### Experiment 2 Training: Color discrimination

The stimulus was the same as in Experiment 1 (see Fig. 1b), except for the stimulus colors. The base color was (*θ*, *s*) = (0, 0.81) for the 0° group and (*θ*, *s*) = (180, 0.81) for the 180° group. The chromaticity of the target stimulus varied in the S positive direction for both groups.

The procedure was the same as that in Experiment 1, except that each session consisted of 320 trials. We increased the number of trials to enhance the training effect since the experiment environment (at each observer’s home) was less controlled than in Experiment 1. The first 20 trials were practice trials that we excluded from further analysis. The training lasted for five days, with one session per day, resulting in a total of 1500 responses (and 100 practice responses).

The analysis procedure was the same as the color discrimination training in Experiment 1. The thresholds were normalized by dividing them by the mean across the training days in each observer to focus on the trend.

### Experiment 2 Test 1: Color difference

#### Stimulus

Figure 4a shows an example of a stimulus in Test 1. The stimulus consisted of four circles, forming two pairs: the top-left and bottom-left pair and the top-right and bottom-right pair. Each pair had either chromatic or achromatic colors; the two circles in a chromatic pair had the same luminance of 20 cd/m^2^ but different chromaticity, while the two circles in an achromatic pair had the same chromaticity of D65 but different luminance. The mean color of the two chromatic circles was one of four colors: saturation *s* = 0.50 at hue *θ* of 0, 90, 180, or 270°. These mean colors are referred to as “base colors.” The chromaticity of each circle deviated from the base color by ± 0.05, ± 0.075, ± 0.10 or ± 0.15 in the cardinal direction roughly corresponding to the hue change direction (e.g., the S direction for the 0° base color and the L-M direction for 90° base color). This degree of hue change is called the “change level.” For example, the pair colors with the base color 0° and the change level of 0.05 had chromaticities of (L-M, S) = (0.50, 0.05) and (L-M, S) = (0.50, − 0.05). In contrast, the mean luminance of the two achromatic circles was 25 cd/m^2^, which is referred to as “base luminance.” The luminance of each circle deviated from the base luminance by ± 1.0, ± 1.5, ± 2.0 or ± 3.0. Thus, all the circles had higher luminance than the background (20 cd/m^2^). These color pairs are summarized in Table 4. As can be seen from Table 4, there were a total of 20 color pairs. Since we created each stimulus by combining two color pairs for Thurston’s pairwise comparison, there were $$\left(\begin{array}{c}20\\ 2\end{array}\right)=190$$ different stimuli in total.

**Table 4:** Color pairs in Test 1 of Experiment 2.

Chromatic pair		Achromatic pair	
Parameter	Details	Parameter	Details
Base color hue *θ* (°)	0, 90, 180, 270	Base luminance (cd/m^2^)	25
Change level	0.05, 0.10, 0.15, 0.20	Change level (cd/m^2^)	1.0, 1.5, 2.0, 3.0

#### Procedure

We measured the relative perceptual color differences of the 20 stimulus pairs using Thurston’s pairwise comparison method. In each trial, two of the 20 circle pairs in Table 4 were presented side-by-side for 500 ms, followed by a fixation point on a background screen. The position of the colors (top or bottom) of each circle pair was randomized in each trial. The observer indicated which pair (left or right) had a greater color difference using the keyboard in a 2AFC manner. The next stimulus appeared 300 ms after the response. Each session consisted of 190 trials, and two sessions were run in total. The order of the circle pairs was randomized within a session. A message was displayed after completing half of the trials in a session to remind the observer to take a break, and the observer could rest as long as they wanted. No practice trials were provided.

#### Analysis

Preference scale values were calculated from the responses gathered across all observers. In this calculation, we used the z-scores of response probabilities based on Thurston’s Case V model and obtained initial preference scale values. Then, we refined these values using the maximum likelihood method to correct for the bias caused by response probabilities of 0 or 1. Finally, the preference scale values in each of the pre- and post-training results were normalized by subtracting the average across all chromatic pairs except the achromatic pairs to see relative color differences across the chromatic pairs.

### Experiment 2 Test 2: Color category boundary

The stimulus and procedure were the same as Test 1 of Experiment 1 except for the category boundaries to be judged; hue angles corresponding to the six boundaries (pink-purple, purple-blue, blue-green, green-yellow, yellow-orange, and orange-pink) were measured in this experiment. These six boundaries were measured in separate sessions, and the order of the six boundaries was counterbalanced across observers to avoid any order bias. Each observer performed one session with 100 trials for each category boundary. The analysis procedure was also the same as Test 1 in Experiment 1.

### Experiment 2 Test 3: Unique hue

The stimulus and procedure were the same as Test 2 of Experiment 1, except that all of the four unique hues (unique red, unique blue, unique green, and unique yellow) were measured. These four boundaries were measured in separate sessions with 100 trials. The order of the four boundaries was counterbalanced across observers to avoid any order bias. Each observer performed one session for each unique color. The analysis procedure was also the same as Test 2 in Experiment 1.

### Experiment 3: General methods

#### Overall flow of experiment

The experiment followed the test-training-test paradigm with the same schedule as in Experiment 2. The only difference from Experiment 1 was the test phase; we measured the color appearance by a modified elementary color naming and the category boundaries by a categorical color naming.

#### Observers

A total of 12 Tokyo Tech undergraduate and graduate students participated in the experiment as observers (4 females, age range 21–24 years). None of them had participated in Experiments 1 and 2. All observers had normal or corrected-to-normal visual acuity and passed the Ishihara color test. The observers were divided into two groups based on the base color of color discrimination training: 0° group (6 observers) and 180° group (6 observers).

The Ethical Review Committee of Tokyo Institute of Technology approved all the experiments in this study, which followed the Declaration of Helsinki. All observers gave written informed consent.

#### Apparatus

The apparatus was almost the same as Experiment 2. The experiment was conducted at the observers’ homes in the training phase and in the dark room of the laboratory in the test phase.

### Experiment 3 Training: Color discrimination

The methods were the same as Experiment 2.

### Experiment 3 Test: Elementary color naming and categorical color naming

#### Stimulus

The color space and spatial layout of the stimulus were the same as in Experiments 1 and 2 (see Fig. 1b). The four circles had the same color selected from the 36 colors on the hue circle at 10° steps (from 0° to 350°).

#### Procedure

The observer performed the test tasks before and after the color discrimination training. The observer performed both the elementary color naming and categorical color naming successively for each stimulus presentation. In each trial, four circles with one of the 36 colors were displayed. First, the observer reported the appearance of the hue in a two-step procedure as follows. In the first step, the observer selected two hues from the basic four hues (red, blue, green, and yellow) that constituted the color appearance by clicking the mouse button twice: first red or green, then blue or yellow. In the second step, a dark-gray scale bar appeared below the stimulus, and the observer used the visual analog scale on the scale bar to indicate the proportion of the two hues in the color appearance; the scale was adjusted by moving the mouse, and the response was determined by holding a mouse button. Only perceived hues were measured since the stimulus color was controlled only on the hue circle. Then, the observer named the stimulus color using categorical color naming; specifically, they used the numeric keypad to pick the best color name from the six basic color terms of Berlin and Kay^24^ (green, yellow, blue, purple, pink, and orange, which existed on our color stimuli). Then, five colors were randomly selected from a whole hue circle with *s* = 0.81 and presented for 0.1 s each to prevent adaptation to the stimulus color. The next trial began 0.3 s after the response of categorical color naming, and the stimulus appeared again. The observer repeated three trials for each of the 36 colors and completed all the trials in one session. The order of stimulus colors was randomized.

#### Analysis

The responses in the elementary color naming task were analyzed as follows. First, the responses of all observers were summed up, and the ratios of four hue components were calculated, as shown in Fig. 8a. Then, the hue angle of each color in the perceptual opponent color space (0°, 90°, 180°, and 270° correspond to red, blue, green, and yellow) from the vector sum of the four color components in the observer responses; for instance, if the blue and green response ratios were the same for a single color, the hue angle would be 135°. Figure 8b,c show the perceptual hue angle as a function of the stimulus hue angle on the MB chromaticity diagram before and after the training for the 0° and 180° groups, respectively. However, because the elementary color naming results were somewhat variable, and the perceptual hue is unlikely to change abruptly, a five-point moving average was applied along the horizontal axis in these charts. Then, on these charts, we subtracted the perceptual hue angles before training from those after training for each MB hue angle. The resultant values could be considered as the color appearance shift due to training.

The responses in the categorical color naming task were analyzed as follows. First, we assigned one of the basic color terms that received the highest response rate from all observers to each of the 36 colors; this term was referred to as the “category” of this color. Then, we determined the category boundary based on the assigned color categories.

### Supplementary Information

Supplementary Information.

## Data Availability

The datasets generated for this study are available on request to the corresponding author.
