# Supplementary material for: Color discrimination repetition distorts color representations
Source: Sci Rep. 2024 Apr 26;14:9615. doi: 10.1038/s41598-024-60283-4 (PMC11053157; doi:10.1038/s41598-024-60283-4)
Supplement: Supplementary file 1 — Supplementary Information. [file 41598_2024_60283_MOESM1_ESM.pdf]

Supplementary materials

Figures S1 and S2 show the results of Test 1 and Test 2 in Experiment 1. Although the data is the same as Figure 2, the pink-purple boundary, orange-pink boundary, and unique red are plotted in separate panels for the visibility of individual observers' data.

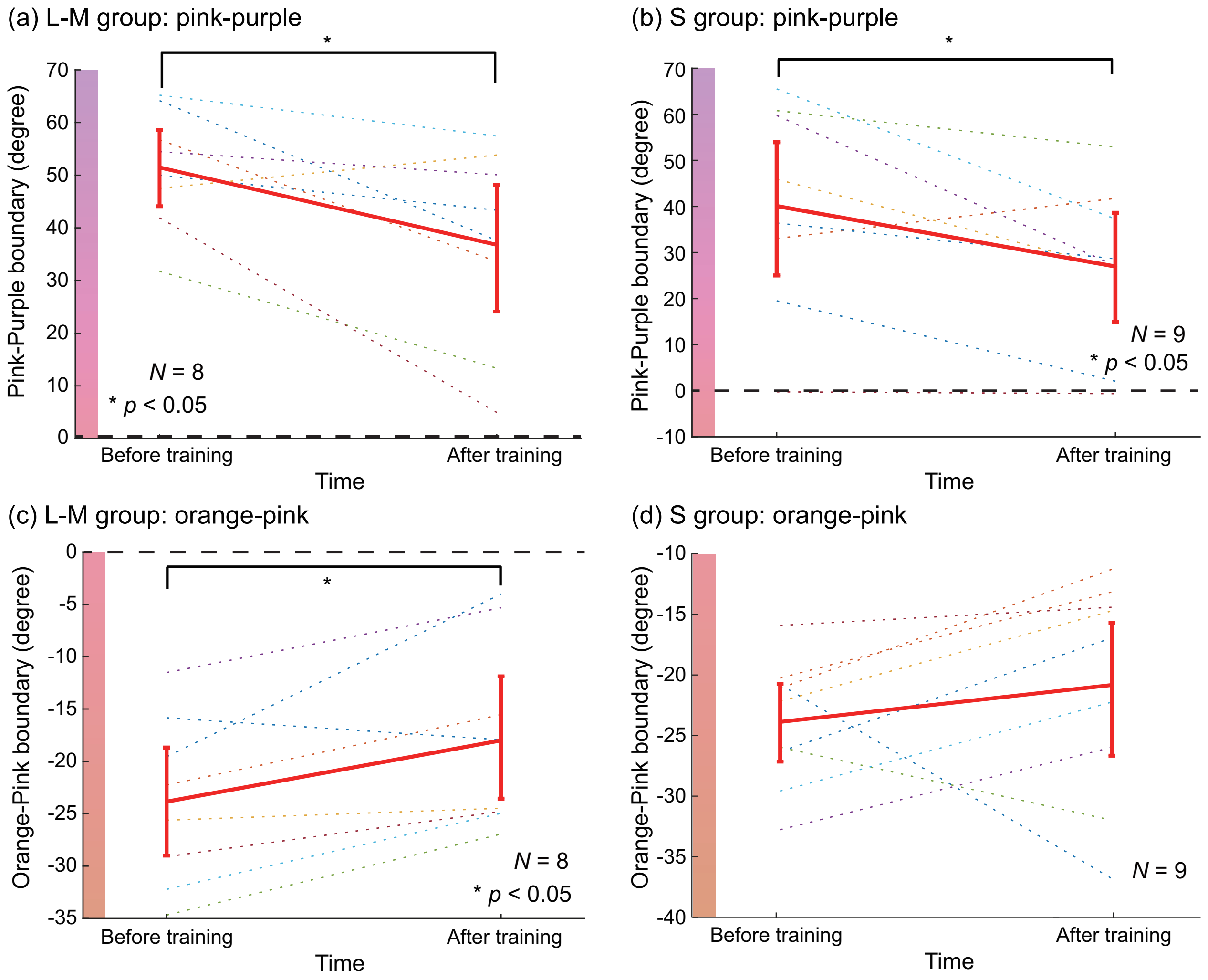

Figure S1. Color category boundaries measured in Test 1 of Experiment 1. (a) pink-purple and (c) orange-pink boundaries in the L-M group and (b) pink-purple and (d) orange-pink boundaries in the S group. The horizontal axis shows before or after training, and the vertical axis shows the hue angle. The colored dotted lines represent the results for individual observers, and the solid red line represents the mean across all observers. The black dotted line shows the trained color of 0°. Error bars indicate 95% confidence intervals obtained from the bootstrap procedure.

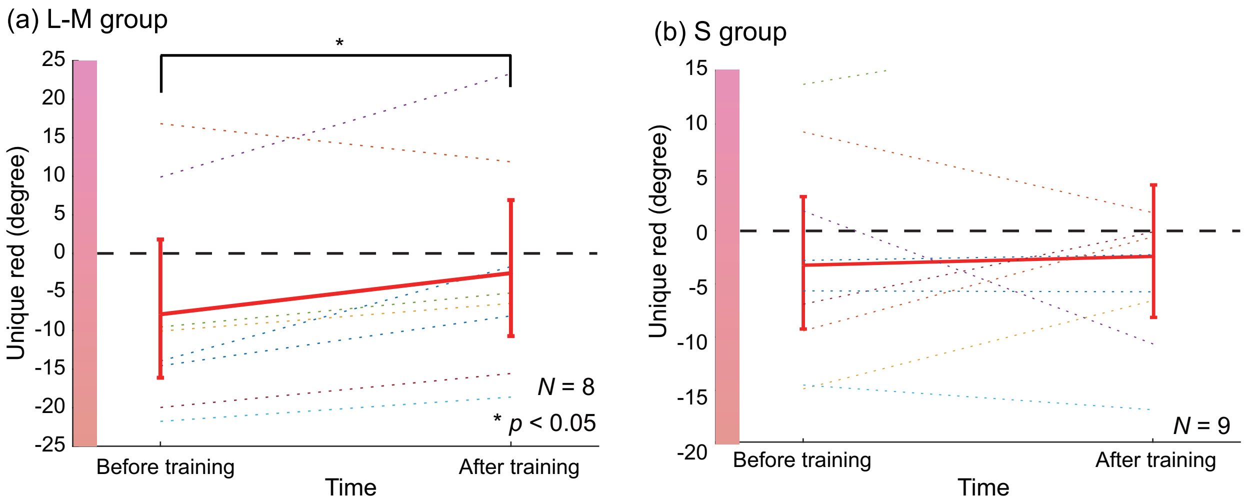

Figure S2. Unique red before and after color discrimination training measured in Test 2 of Experiment 1 for (a) L-M and (b) S groups. The format of each panel is the same as Figure S1 (a) except that the vertical axis indicates the hue angle corresponding to unique red.

Figure S3 shows the perceived color difference at different change levels measured in Test 1 of Experiment 2. In all base colors, the perceived color difference monotonically increased with the change level.

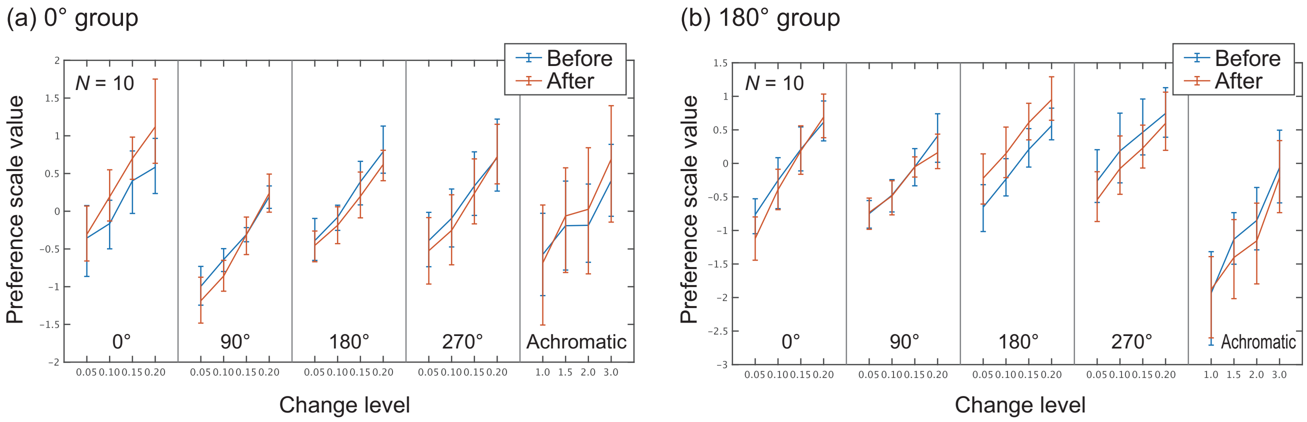

Figure S3. Perceived color difference at different change levels measured in Test 1 of Experiment 2 for (a) 0° and (b) 180° groups. The horizontal axis indicates the base color and change level. The vertical axis shows the preference scale value as an index of relative perceived color difference. The blue and red lines show the results of before- and after-training, respectively. The error bars are the 95% confidence interval obtained from the parametric bootstrap procedure with 10,000 repetitions.

Figure S4 shows the shift in the perceptual hue angle before and after training measured in the elementary color naming in Experiment 3. Although the experimental data was identical to Figure 8 (d) in the main text, the center hue angle of the fitted derivative Gaussian was also included as a free parameter, along with the amplitude and width. The results showed

that the center position was unstable in the 0° group, whereas in the 180° group, the 95% confidence interval of the center position estimated by the bootstrap procedure was 176.3°-212.6°, which was close to the training color. Moreover, the amplitude was significantly negative (that is, the perceived color shifted towards the larger (smaller) hue angle after training when the stimulus color had a larger (smaller) hue angle than the center hue angle of the fitted derivative Gaussian). This finding indicates, as in Figure 8 (d), that the difference in the color appearance around the training color is enhanced after the training.

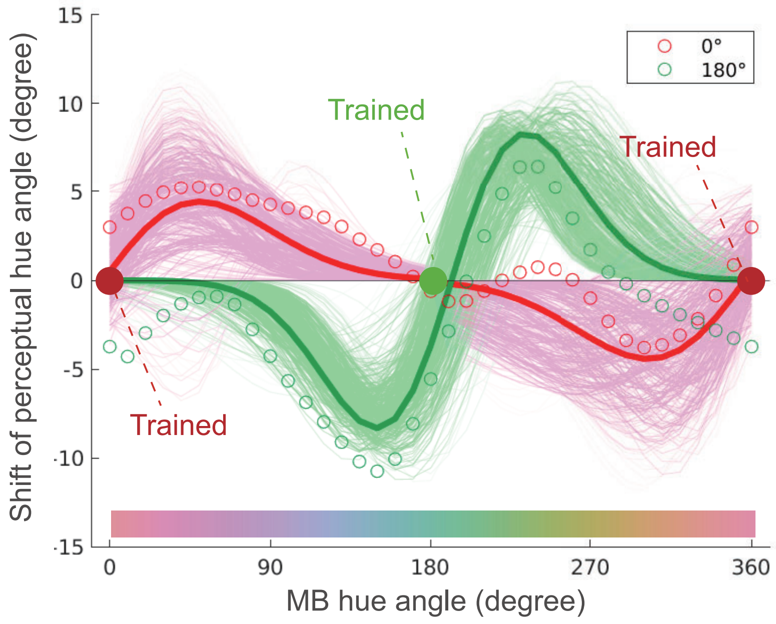

Figure S4. Shift in perceptual hue angle before and after color discrimination training as a function of MB hue angle. The format and data is the same as Figure 8 (d), except that the fitted derivative Gaussian had three free parameters: amplitude, width, and center hue angle.

Tables S1 and S2 show the intended and measured luminance and chromaticity for several stimulus colors on the displays in the darkroom for Experiments 1 and 2, respectively. Table S3 shows the chromaticity and luminance of the laptop for Experiment 2 in the relatively dark room, which is covered by a curtain during the day. The luminance and chromaticity were measured using the spectroradiometer.

Table S1. Intended and measured luminance and chromaticity in Experiment 1.

Hue angle (degree)	Intended color			Measured color		
	Luminance	*u'*	*v'*	Luminance	*u'*	*v'*
Background	20.00	0.2105	0.4737	17.2	0.2112	0.4753
0	19.68	0.2954	0.4748	16.7	0.2963	0.4640
45	19.57	0.2713	0.4438	16.6	0.2727	0.4308

90	19.62	0.2159	0.4375	16.8	0.2167	0.4240
135	19.79	0.1556	0.4579	17.0	0.1576	0.4464
180	19.98	0.1202	0.4972	17.1	0.1215	0.4875
225	20.09	0.1361	0.5353	17.1	0.1389	0.5289
270	20.04	0.2009	0.5448	17.4	0.2027	0.5425
315	19.87	0.2693	0.5173	17.1	0.2715	0.5120

Table S2. Intended and measured luminance and chromaticity in Experiments 2 and 3.

Hue angle (degree)	Intended color			Measured color		
	Luminance	*u'*	*v'*	Luminance	*u'*	*v'*
Background	20	0.2105	0.4737	18.3	0.2079	0.4783
0	19.75	0.2458	0.4805	17.9	0.2427	0.4747
45	19.62	0.2380	0.4510	17.7	0.2353	0.4431
90	19.62	0.2155	0.4420	17.8	0.2140	0.4336
135	19.73	0.1897	0.4569	18.0	0.1891	0.4499
180	19.90	0.1733	0.4898	18.0	0.1732	0.4840
225	20.02	0.1776	0.5241	18.2	0.1768	0.5201
270	20.03	0.2029	0.5364	18.2	0.2013	0.5337
315	19.91	0.2322	0.5168	18.0	0.2297	0.5128

Table S3. Intended and measured luminance and chromaticity in Experiments 2 and 3 in rather bright environment.

Hue angle (degree)	Intended color			Measured color		
	Luminance	*u'*	*v'*	Luminance	*u'*	*v'*
Background	20	0.2105	0.4737	21.1	0.2079	0.4773
0	19.75	0.2458	0.4805	20.6	0.2426	0.4736
45	19.62	0.2380	0.4510	20.2	0.235	0.4418
90	19.62	0.2155	0.4420	20.4	0.2138	0.4325
135	19.73	0.1897	0.4569	20.7	0.1893	0.4486
180	19.90	0.1733	0.4898	20.6	0.1735	0.4829
225	20.02	0.1776	0.5241	20.8	0.1772	0.5191
270	20.03	0.2029	0.5364	20.7	0.2009	0.5330
315	19.91	0.2322	0.5168	20.6	0.2297	0.5119
